# Cooperation networks of ambulatory health care providers: exploration of mechanisms that influence coordination and uptake of recommended cardiovascular care (ExKoCare): a mixed-methods study protocol

**DOI:** 10.1186/s12875-020-01229-3

**Published:** 2020-08-16

**Authors:** Christine Arnold, Patrick Hennrich, Jan Koetsenruijter, Jan van Lieshout, Frank Peters-Klimm, Michel Wensing

**Affiliations:** 1 Department of General Practice and Health Services Research, Heidelberg University Hospital, Im Neuenheimer Feld 130.3, 69120 Heidelberg, Germany; 2 Department IQ healthcare, Radboud Institute of Health Sciences, Radboud University medical center, P.O. Box 9101, 114, 6500 Nijmegen, HB The Netherlands

**Keywords:** Coordination of care, Social network analysis, Cardiovascular diseases, Ambulatory cardiology care, Germany, Cooperation

## Abstract

**Background:**

As the number of elderly and multimorbid patients increases, healthcare has become more complex. This requires good coordination of treatment and care given the various  health care professionals involved (e.g. general practitioners, medical specialists, physicians’ assistants). Lack of coordination jeopardizes seamless, evidence-based treatment and care, and eventually reduces clinical effectiveness. The aim of the study is a) to describe and explore information transfer and interprofessional collaboration in ambulatory cardiac care, b) to describe and explore the role of provider networks from the perspective of patients and providers, focusing on healthcare coordination and the uptake of recommended practices.

**Methods:**

Two related studies are planned: a) an observational study of healthcare provider networks, involving 600 patients with chronic (atherosclerosis-related) cardiovascular disease from 40 general practices and up to 320 healthcare providers (general practitioners, medical specialist, physicians’ assistants), and b) a qualitative interview study with up to 80 healthcare professionals and patients. Furthermore, we will analyse claims data of a large German health insurer to explore provider networks in ambulatory cardiac care.

**Discussion:**

The project aims to provide insight into factors, processes and mechanisms of information transfer and interprofessional collaboration, which affect seamless, evidence-based healthcare practice. This will contribute to the design of strategies for improving health care practice and to the development of measures of coordination for future research.

**Trial registration:**

We registered the study prospectively on 7 November 2019 at the German Clinical Trials Register (DRKS, www.drks.de) under ID no. DRKS00019219.

## Background

Health outcomes can only be optimized when recommended treatment and care are implemented comprehensively in healthcare practice, and unnecessary or unsafe practices are avoided. This can be enhanced by a variety of strategies, such as continuing medical education of healthcare professionals (HCP) and financial incentives for healthcare providers. Implementation strategies need to address the fact that increasing numbers of patients receive healthcare from a range of HCPs, simultaneously or consecutively, as a result of increasingly complex care needs and ongoing specialization of healthcare providers. These professionals have different backgrounds (in medicine, nursing and allied health professions) and are based in various healthcare organizations and sectors (e.g. ambulatory care, hospitals, rehabilitation centres). Their involvement in patient care may fluctuate over time; for instance, before, during and after hospital admission. In some settings, HCPs collaborate in structured teams, but such patient care teams (with regular meetings to share information and coordinate activities) are uncommon in ambulatory care and across primary and hospital care. Although several health professionals are usually involved in the care for a patient, they do not necessarily collaborate actively. In those situations, it is a challenge to achieve well-coordinated healthcare delivery; healthcare which is clinically coherent and logistically non-interrupted over time and across providers. Low coordination is associated with increased risks for patient safety [1], lowered patient activation [2], avoidable hospital admissions [3], and lowered patient experience of confidence and safety [4]. The German healthcare system is (like other modern healthcare systems) fragmented, and achieving better coordination of care is high on the health policy agenda [5].

Coordination of care is influenced by many factors. Firstly, shared ideas about what is desirable practice, such as shared multi-disciplinary clinical guidelines, enhance coordination across different healthcare providers. It is difficult to achieve well-coordinated care, if each healthcare profession and institution follows its own recommendations and these are conflicting with those of others. Secondly, coordination depends on effective cooperation between healthcare providers around individual patients and local populations. This requires adequate information exchange, constructive cooperation behaviours, and good relationships between patients and providers [6]. Cooperation work on a case-by-case basis is time consuming, so much of it takes the format of structured information transfer (e.g. referral letters of general practitioners to other medical specialists). HCPs’ attitudes and competencies influence their interprofessional cooperation. Third, a range of contextual and health system factors (financial, organisational, legal) are barriers or facilitators of well-coordinated care. For instance, if coordination implies additional workload or a financial loss, which is not compensated for, it is unrealistic to achieve improvement in practice.

The proposed study will explore the role of population structure [7], particularly emerging social networks of healthcare providers [8], on healthcare providers’ performance and patients’ experience of healthcare. For this purpose, we will apply social network analysis (SNA) to examine healthcare providers’ cooperation. The usefulness and feasibility of SNA methods were successfully tested in pilot studies [9–11]. The aim of the study is a) to describe and explore variation in information transfer and interprofessional collaboration in ambulatory cardiac care in Germany and b) to describe and explore the role of provider networks from the perspective of patients and providers, focusing on healthcare coordination and the uptake of recommended practices.

## Methods and design

The research project ‘ExKoCare’ is a multicentre, observational (non-interventional) study that uses a mixed-methods approach including a) a quantitative survey study, b) a qualitative interview study and c) a quantitative analysis of health insurance claims data.

### Quantitative survey study

#### Design and setting

Observational study in a stratified random sample of 40 general practitioners’ practices (GP practices) and surrounding cardiologists (approximately 5 per GP practice) with whom they are likely to collaborate regarding the targeted patient populations. The sample will involve practices from three different German states (Baden-Württemberg, Rhineland-Palatinate and Saarland). If the numbers allow for it, the sample will be stratified such that it adequately represents small and large practices, practices within and outside of organized practice networks, practices within and outside of GP-centred care (“Hausarztzentrierte Versorgung”), and practices in rural as well as urban areas. As these characteristics are not all available upfront (except for training practices), we will recruit a random sample and sample within strata to compose the stratified sample. For practical reasons, we intend to recruit within an area of maximum 200 km from Heidelberg.

#### Study population and sampling

From publicly available lists of the Statutory Health Insurance Agencies of Baden-Württemberg (KVBW), Rhineland-Palatinate (KVRLP) and Saarland (KV Saarland), primary care practices within designated administrative districts will be randomly drawn and invited to take part in the study until the target sample size has been reached.

To enhance the homogeneity of the study population, we will focus on patients with coronary heart disease (as indicator for atherosclerotic vascular disease) and at least one other chronic disease (chronic heart failure, diabetes mellitus, chronic obstructive pulmonary disease, chronic renal failure or depression). Within these conditions, we will select patients with moderate to high disease severity, using ICD-codes. Primary care practices will be asked to invite 15 patients for the study. Primary care physicians, respectively their assistants, will be asked to generate a list of patients potentially eligible for our study by using their practice’s documentation software. Patients need to have visited the practice within the past 3 months; patients with a short life expectancy or major cognitive limitations will not be approached. The practice generates a printed list and removes potential identifiers except ICD-codes. Afterwards, one member of the research team visits the practice and randomly chooses the desired number of patients from the list. Alternatively, instructions for randomized sample drawing are sent to the practice and staff members choose patients accordingly themselves. The practice is then provided with a sufficient number of blank envelopes and questionnaires through the research team and will send out questionnaires to the selected patients. The patient receives the questionnaire, is free to fill it out and send it back anonymously directly to the research institute. The study was powered to detect a small to medium effect size (*r* = 0.15) on the primary outcome. Power was set at 0.8, significance level at 0.05, and a design effect of 1.4 (40 clusters and an intraclass correlation coefficient of 0.01) was taken into account.

Ambulatory cardiologists will be contacted based on the geographical area they are based in. Depending on the location of the primary care practices in the study, cardiologists within a radius of about 25 km will be invited to take part in the study. They will be identified by publicly available data from the online database of the respective Statutory Health Insurance Agency.

#### Primary and secondary outcomes

The primary outcome is patient-reported coordination of healthcare. Coordination of patient care is a multidimensional concept, often measured in terms of patient reported healthcare received [12]. For this study, a validated patient questionnaire will be applied: the Nijmegen Continuity Questionnaire, a 28-item questionnaire that covers personal coordination of care as well as multi-professional cooperation [13]. The measure has been applied and validated in primary care as well as ambulatory medical specialist care. It has been translated into German, using a forward and backward translation procedure, and pilot tested in a separate sample of patients before the main study began. The 4 items concerning inpatient care will not be used.

The main measures in this study map out the cooperation networks of healthcare providers.

The patient questionnaire will contain questions on healthcare providers involved and their cooperation up to 1 year before measurement. This method was successfully applied in previous research of the principal investigator [11]. The focus of the questionnaire will be on ambulatory cardiac care (primary care physician, physicians’ assistants, cardiologist). Based on previous research of the principal investigator [14], we refrain from measuring networks from the patient relatives’ perspectives.

Additionally, several network characteristics will be calculated from the documented cooperation networks (centrality, density, homogeneity, reciprocity: see section data-analysis), while other network characteristics will be directly measured with questionnaires for providers (opinion leadership, presence of case managers, perceived change of networks). The questionnaire for primary care providers will include questions on the social network and its context: a) presence of opinion leaders for vascular care [14, 15] within and outside of general practices as mentioned by health care providers. Furthermore, b) providers’ self-perception of opinion leadership [15, 16], c) clinical attitudes regarding treatment of vascular patients on which controversy exists, using validated questions with a 5-point Likert scale [14], d) attitudes regarding interprofessional cooperation, using a validated questionnaire [17], e) identification of an appointed case manager for vascular care, if present and f) perceived change in the primary care practice and in the regional network for cardiovascular care (low, moderate, high). We will also include open questions to identify contextual determinants of cooperation between healthcare providers, including factors related to the functioning of primary care teams, financial reimbursement schemes, information technology systems, and health system characteristics.

#### Questionnaires for patients, health care providers and practices

All questionnaires will be sent out after inclusion of the practices and within the first 12 months of the project.

Patients‘questionnaires (see Additional file 4) will include questions on sociodemographic patient characteristics (6 questions), (multimorbid) diseases (2 questions) and disease management programs (2 questions) using standardized questions. Opinions on healthcare providers and healthcare provision by general practitioners and medical specialists in cardiology will be collected through the Nijmegen Continuity Questionnaire described above (24 questions). Additionally, patients will be requested to report on the frequency of contacts with their own GP, cardiologists (6 questions) and other healthcare providers such as physicians’ assistants, other medical specialists, groups for cardiovascular-related sports, pharmacies, podiatries and home care services (1 question). Finally, the patient questionnaire will document aspects of cardiac care delivery, derived from prevailing clinical guidelines (e.g. treatment and counselling received, 10 questions).

Primary care practices will receive a questionnaire on background characteristics of the practice itself (6 questions), staff and education (5 questions), documentation (1 question) and communication with medical specialists (1 question), see Additional file 3.

Primary care providers (physicians, assistants) (see Additional file 2) will complete a questionnaire on sociodemographic data (4 questions), professional career (3 questions), interaction with staff (personalized, 2 questions, similar to a measure used in previous research [10]) and cardiologists (personalized, 2 questions), communication and information exchange with cardiologists (4 questions) as well as information exchange with pharmacists, nutritionists, physiotherapists, elderly care nurses, ambulatory care services, sport groups for rehabilitation patients, sport groups for cardiovascular patients, facilities for rehabilitation, other general practitioners and other practice assistants, psychologists, pneumologists, specialists for internal medicine and medical officers (2 questions, a similar measure was successfully applied in previous research of the principal investigator [10, 14]). Furthermore, they will be asked about their opinion on cardiovascular care and whether they see themselves more as opinion leaders or opinion seekers (16 questions). Finally, they receive questions on team climate within their practice (15 questions). A validated 14-item questionnaire will be used: the Team Climate Inventory, short version [17], and one additional question on factors that could influence health care delivery (1 question).

Physicians’ assistants will receive the same questionnaire and are asked to skip questions explicitly related to physicians.

Cardiologists will be requested to complete a short, written questionnaire (Additional file 1). We will ask sociodemographic and professional characteristics (6 questions) and questions on information exchange with other professionals, such as: general practitioners, pharmacists, nutritionists, physiotherapists, elderly care nurses, ambulatory care services, sport groups for rehabilitation patients, sport groups for cardiovascular patients, facilities for rehabilitation, other practice assistants, psychologists, pneumologists, specialists for internal medicine except cardiology and medical officers (2 questions, see above). Furthermore, they will be asked about their opinion on cardiovascular care (3 questions) and factors influencing health care delivery (1 question).

#### Data-analysis

##### Phase 1. Data-verification

Item response and frequencies of all questionnaire data will be inspected and explored in order to identify selective item-response. Within primary care practices and the local networks of primary care and cardiology practices, the overlap of reported cooperation networks across responders will be examined to get an impression of the reliability and completeness of network data. Reciprocity of reported connections is used as an indicator of reliability of data on presence of connections. If the reciprocity is sufficiently high (> 0.60), missing values will be imputed by available data [18].

##### Phase 2. Construction of cooperation networks

Two networks will be constructed for each of the 40 primary care practices. The first network is provider-reported. Data from primary care providers in a specific practice organisation will be combined in one data-set and links with healthcare providers outside the practice (cardiologists and other providers) are added. The second network is patient-reported. Data from individual patients in a specific practice organisation will be combined in one data-set in order to define the cooperation network from the patient perspective. Identifiers of individuals in the provider- and patient-based networks will be matched in order to examine the overlap between the two types of networks.

##### Phase 3. Descriptive data-analysis (research objective 1)

Data preparation will be done with the statistical package SPSS, while descriptive and comparative analyses will be made using software packages SPSS and R. The measured aspects of coordination and their variation between practices will be displayed descriptively, using suitable summary statistics. The general practices’ networks will be visually displayed in network plots using visualization within R. Descriptive network parameters (size, density, centrality) will be presented, including mean, median, and range of values. Overlap between patient and care provider reported networks will be calculated using correlation/multiplicity measures. Descriptive and visual information will be reported to participating practices as educational feedback.

##### Phase 4. Exploration of network-related determinants of coordination (research objective 2)

Quantitative analysis is primarily focused on the hypotheses concerning network-related mechanisms of coordination, which are specified in the conceptual model.

The respective mechanisms will be examined in two steps:

Step 1: Construction of measures for each practice, calculated from the documented networks or directly from the questionnaires:

- opinion leader: presence of a care provider within or outside of a general practice influencing opinions as reported by care providers. (polytomous variable). Additionally, self-perception as an opinion leader by care providers (based on questionnaire).
- central care provider: degree of the healthcare provider with the highest centrality in the network (count variable, calculated from network data).
- density of network: proportion of connections of all possible connections (variable with values between 0 and 1, calculated from network data).
- homogeneity: consistency of clinical attitude views and multiprofessional cooperation (E-I parameter, which has values between − 1 and + 1, calculated from network data).
- presence of case manager (dichotomous variable, based on questionnaire).
- network reciprocity: average proportion of network members which can be reached through mutually confirmed pathways (count variable, calculated from network data).
- perceived change in the network of referral and consultation to other care providers (5-point-likert-scale, based on questionnaire).

Step 2: Analysis of impacts. Potential impact of each of the network characteristics together with patient-reported coordination of care will be examined. The measured and constructed network characteristics will be added to the patient-level data as potential predictors for the patient-reported coordination of care and aspects of cardiac care delivery. We will analyse the provider-reported cooperation networks and the patient-reported cooperation networks separately. Linear or logistic regression models will be applied with distributions and link functions as appropriate for the observed distributions (e.g. normal, binomial, Poisson). Random coefficient models will be used in order to take clustering of data into account (patients with practices). The influence of individual patient characteristics will be explored in further analyses. Although a large number of statistical tests will be applied, we use the conventional *p* < 0.05 as cut-off for significance given the explorative character of the study.

### Qualitative study

#### Design and setting

The research project also comprises a qualitative interview study in the complete sample of practices, which was recruited for the quantitative study. The interviews with representatives in all practices aim to contextualize the role of network-related mechanisms of coordination in a broader range of influences. A purposeful subsample of low and high performing practices will be examined to explore hypotheses on network-related mechanisms. Focus group interviews with stakeholders and experts will be done to explore options for strategies that relate to cooperation networks.

First, representatives (usually a GP) of all 40 participating practices will be interviewed. Second, a purposeful sample of other individuals from these practices will be examined in more detail. Maximum variation will be sought by targeting 4 practices with high scores for coordination and implementation of recommended practices and 4 practices with low scores on these measures. In each of these practices, we aim to interview face-to-face or via telephone (depending on the interviewees’ preferences) three patients, a primary care provider, and another healthcare provider (e.g. physician or physician’s assistant). The targeted sample size for this study is therefore 40. Finally, an independent purposeful sample of 10 to 15 stakeholders in the German healthcare system and experts in healthcare innovation will be interviewed and involved in the interpretation of study findings.

#### Study population and sampling

Representatives of primary care practices in the quantitative study phase will all be invited to participate in this interview study. Recruitment of the subsample will be carried out separately after completion of the necessary calculations regarding coordination and implementation. Here, physicians and practice staff of the selected practices will be invited to participate. Regarding patient interviews, new patients will be randomly selected and invited to take part.

#### Interviews

Interviews with representatives of practices will be semi-structured and cover a range of potential determinants of healthcare coordination, such as cultural factors, financial consequences, and patient expectations. This will help to interpret the role of network-related factors in relation to other determinants of healthcare coordination. Semi-structured interviews, organised by a pilot-tested interview guide and conducted by trained interviewers, will focus on network-related determinants of coordination. Besides exploration of the hypothesised mechanisms, the interviews will also contain open questions to identify further factors and mechanisms related to networks.

Focus group study. Based on earlier research of the principal investigator [19], we will organize focus groups (6–8 participants per group) led by trained facilitators to identify potential strategies that build on network-related factors to improve healthcare coordination. In the group meetings, key information from the research will be presented, followed by a brainstorm among participants.

All interviews will be audiotaped and fully transcribed by trained staff. Data will be kept confidential and pseudonymized in a secured place, with the key to personal identifiers in a separate place; personal identifiers will be destroyed after use.

#### Data-analysis

The qualitative study relates to research objective 2, which concerns network-related mechanisms of information exchange and collaborative work in the healthcare setting. The study aims to provide insight into patterns of context, mechanisms, and outcomes [20]. It will particularly explore which social network characteristics influence mechanisms of information exchange and collaborative work in healthcare delivery networks.

**Fig. 1:**
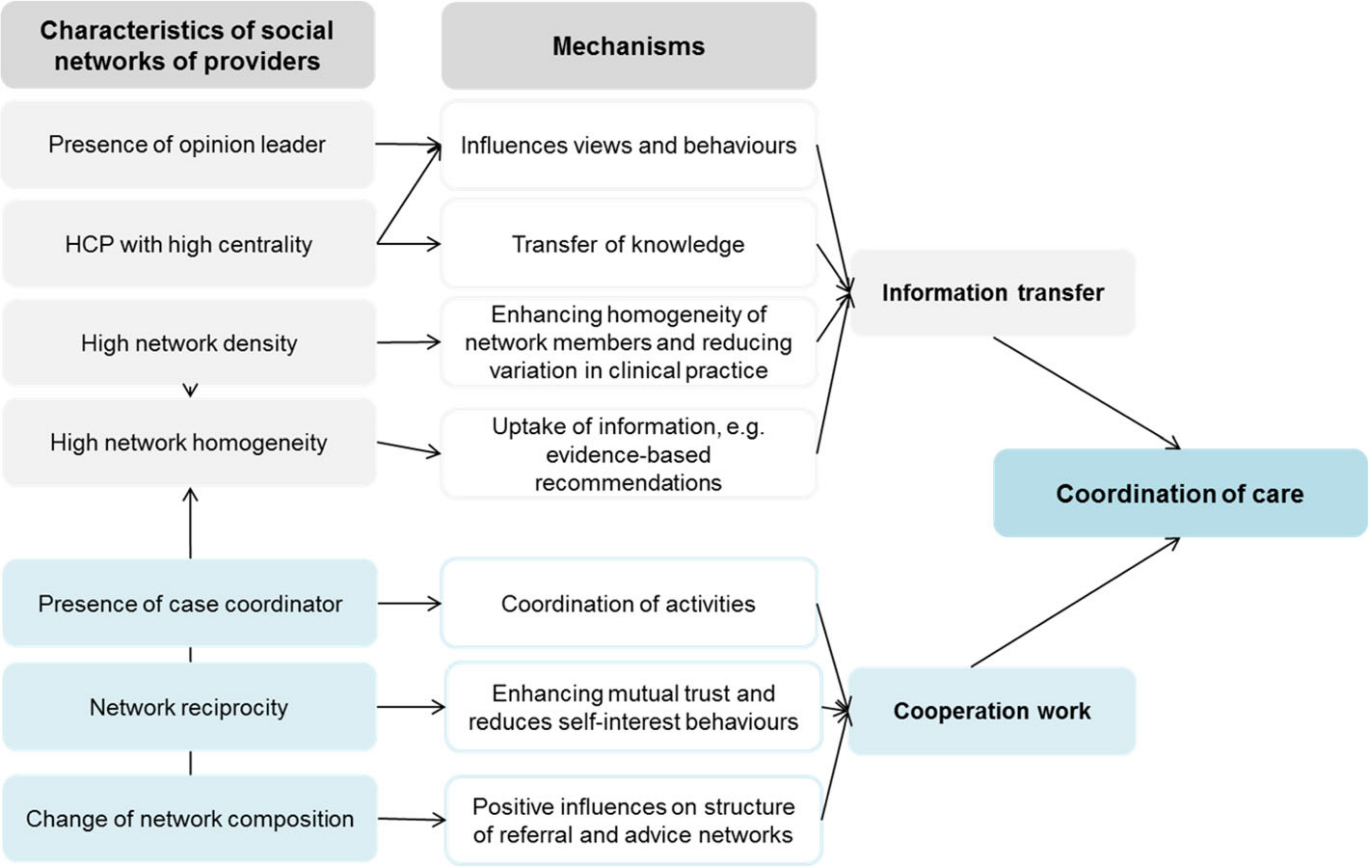
Conceptual model showing hypothesized mechanisms affecting coordination of care

For organizing the steps in the data-analysis, we will apply the framework method, which is a structured, stepwise approach that centres around a matrix of cases (usually participants) in rows and interpretations (codes, categories, etc.) in columns [20]. In this way, codes and categories remain linked to cases. The framework method is not inherently deductive or inductive, and we will use both. In the deductive approach, we will use the conceptual framework for network-related factors of healthcare coordination, which was developed for this proposal (Fig. 1), and a comprehensive framework of 57 conceptual determinants of implementation of practices into healthcare practice, which has been systematically developed in previous research of the principal investigator and has proven to be useful in a range of studies [19].

Following published guidance [21], the following stages are planned: After interviews have been fully transcribed (stage 1), the researchers will familiarize themselves with the transcripts by reading these line-by-line (stage 2). Then relevant sections of the transcripts will be coded: descriptive or conceptual labels will be attached (stage 3). Following Emerson et al. [22], we will apply open coding first (coding anything that might be relevant from different perspectives). This is followed by focused coding in a fine-grained analysis on the basis of the concepts in the pre-defined frameworks. At least two researchers will be involved in the coding of the initial interviews. They will meet after coding initial interviews in order to develop a working analytical framework, in which codes have been organized in categories (clusters of codes) (stage 4). They will then code additional interviews and meet again; this will be repeated until the framework has been consolidated. The resulting framework will be applied to the remaining interviews (stage 5).

After completion, a spreadsheet is used to create a matrix in which the codes are charted in relation to participants (stage 6). Throughout the data-analysis, reflections on the interpretation of data will be documented in analytical memos and used to generate interpretations in discussion with researchers, patients and clinicians (stage 7). Finally, linkages between the findings of quantitative data-analysis and the results of the qualitative analysis will be explored, using available options for joint visual display of findings from mixed-methods research [23].

Combining two-step analytical coding in the framework analysis [20] facilitates accumulation of scientific knowledge, because it relates findings to available theories and helps to identify factors and mechanisms that are currently not well covered by theories. A team of trained qualitative researchers will conduct the different steps and cross-check the consistency of the thematic analysis and categorization within the iterative process. Software for qualitative data-analysis (MaxQDA) is applied for logistical support. Data saturation is considered reached when no more new aspects can be identified in the interviewees’ statements.

### Claims data study

#### Design and setting

As the third component of the research project, an independent study (not related to the questionnaires or interview) is planned, based on an anonymized set of administrative claims data. These data will be derived from AOK (“Allgemeine Ortskrankenkasse”) Baden-Württemberg, the largest health insurer in the state, covering about 45% of the 11 million inhabitants. By using claims data, real world data is used instead of questionnaire based data, and it allows to include far more participants and thus increase statistical power to be able to detect smaller effects on clinically relevant outcomes. This is especially important for social network analysis in which missing nodes can significantly affect the results. Moreover, a near to full sample from all physicians is achieved, even if only 45% of the population is included in the sample, adding to the generalizability of our study at large.

#### Study population and sampling

The study population will be adults with recorded coronary heart disease and adults with heart failure regardless of date of diagnosis. For practical purposes, the focus is on patients and health providers in Baden-Württemberg only. Patient-Provider contacts for healthcare delivery, whether by face-to-face, telephone or online (excluding contacts for administrative/logistic purposes only, e.g. making an appointment) will be counted (concentration of care with a single provider). Given the source of the data, only financially reimbursed visits, are covered. Also recorded will be all visits to ambulatory providers, including physicians, psychologists, therapists and hospitals. Pharmacies and diagnostic laboratories are excluded given their complementary role in health delivery. These records will be at least 2 years old upon receipt.

#### Measures

Following a previous empirical study, claims data are used to construct two indices to assess the coordination of healthcare: a) the concentration of care with a single provider and b) number of handoffs of information required between providers [24]. The previous study suggested that these records were highly correlated with measures of the degree of collaboration between different providers during an episode and degree of coordination required between different providers during an episode [24]. As a third outcome, we measure hospitalization for chronic cardiovascular disease patients as an indication for adverse effects of suboptimal treatment.

#### Data analysis

Data preparation and analyses using the claims data will be performed with the relational database MySQL and R. In the first phase, network characteristics will be calculated wherever possible. These include independent measures that theoretically could influence our outcomes and involve among others: network density and homogeneity, the existence of a central care provider, and change of network composition. As outcomes, we will calculate measures that assess coordination of healthcare: a) the Bice-Boxerman Continuity of Care Index (COC) [25] that indicates the concentration of care with a single provider and b) the Sequential Continuity Index (SECON) [26] that indicates the number of handoffs of information required between providers. Additionally, a more clinically relevant outcome will be included by calculating hospitalization.

In the second phase, we will explore whether the hypothesised network-related mechanisms relating to central care providers, network density, network homogeneity, and change of network composition are associated with measures of coordination of care and hospitalization rates. All analytic analyses will be adjusted for patient characteristics, such as age, sex, and prevalence of comorbidities.

## Discussion

This study aims to provide insight into factors, processes and mechanisms of information transfer and interprofessional collaboration, which affect seamless, evidence-based healthcare practice. The results will ultimately contribute to the design of strategies for improving health care practice and to the development of measures of coordination for future research.

There is an extensive body of research on the impact of social networks on human behaviours, including collaboration beyond immediate self-interest [7, 8]. Few of the insights emerging from this research are related to healthcare providers, and social network studies in healthcare providers tend to be largely descriptive [27]. The planned research project will essentially test a number of hypotheses, which were derived from the broader non-health literature, and simultaneously explore health-specific processes (such as the content of referral letters). We are uncertain to what extent healthcare professionals and patients recognize and report network-related processes. Previous studies suggested that the range of factors, mechanisms and processes is substantially broader than perceived and reported by patients or providers in interviews [28].

The findings of the study aim to contribute to the design and evaluation of future interventions, particularly programs for improving coordination and enhancing evidence-based practice. We expect that network-related factors can be used to tailor the main intervention according to these factors. For instance, different approaches may be required in regions with well-connected physicians than in regions with loosely connected physicians. It remains to be seen to what extent discrepancies exist between factors that are perceived to be relevant and factors that are found to influence relevant outcomes, but are not perceived as such.

Given the specific features of the German healthcare system, such as medical specialists in ambulatory care practices, it also remains to be seen to what the findings can be generalized to other healthcare systems (or even states within Germany). Beyond exploring the specific issues in one German state, the project aims to provide fundamental insight into healthcare coordination and the impact of networks that emerge from sharing patients on healthcare providers' professional behaviours.

## Supplementary information

**Additional file 1.** Questionnaire_cardiologists.

**Additional file 2.** Questionnaire_GPs_provider.

**Additional file 3.** Questionnaire_GPs_practices.

**Additional file 4.** Questionnaire_patients.

**Additional file 5.** NCQ_german.

**Additional file 6.** COREQ Statement.

**Additional file 7.** STROBE Statement.

## Data Availability

Not applicable.

## Abbreviations

COC: Bice-Boxerman Continuity of Care Index
GP practice: General practitioners’ practices (GP practices)
HCP: Healthcare professionals
ICD-codes: International Statistical Classification of Diseases and Related Health Problems
SECON: Sequential Continuity Index
SNA: Social network analysis
