# Supplementary material for: Cooperation networks of ambulatory health care providers: exploration of mechanisms that influence coordination and uptake of recommended cardiovascular care (ExKoCare): a mixed-methods study protocol
Source: BMC Fam Pract. 2020 Aug 16;21:168. doi: 10.1186/s12875-020-01229-3 (PMC7429883; doi:10.1186/s12875-020-01229-3)
Supplement: Supplementary file 1 — Additional file 1. Questionnaire_cardiologists. [file 12875_2020_1229_MOESM1_ESM.docx]

Questionnaire for medical specialists for cardiology

[translated by the authors from German to English, translation not validated]

# Part 1: General questions

Please check the answer that matches you.

| 1.1 Year of birth | \|___\|___\|___\|___\| | | | |
| --- | --- | --- | --- | --- |
| 1.2 Sex | Female | Male | | Not specified |
| 1.3 What is your activity status?  Please be aware that this includes any paid/income-related work. | Working full time (35 hours per week or more)  Working part time (less than 35 hour per week) | | | |
| 1.4 In which year did you take up residence? (Please insert year) | \|___\|___\|___\|___\| | | | |
| 1.5 You are a medical specialist for: | Internal medicine: | | without focus  with a focus on cardiology | |
|  | Internal medicine and cardiology  Other: ______________________________ | | | |
| 1.6 Do you have additional medical qualifications? | No  Yes, the following:   1. _________________________________ 2. _________________________________ 3. _________________________________ 4. _________________________________ | | | |

Part 2: Questions on information exchange between health care providers

Hereafter, please indicate all occupational groups **outside** of your practice, with which you exchanged information at least once a week on patients with (1) chronic heart failure and (2) coronary heart disease. This exchange encompasses counselling and treating individual patients.

| 2.1 Persons outside of your practice | Chronic heart failure | | | | | Coronary heart disease | | | |
| --- | --- | --- | --- | --- | --- | --- | --- | --- | --- |
| General practitioners |  | | | |  | | | | |
| Pharmacists |  | | | |  | | | | |
| Nutritionists |  | | | |  | | | | |
| Physiotherapists |  | | | |  | | | | |
| Carers for the elderly in a nursing home |  | | | |  | | | | |
| Ambulatory nursing services |  | | | |  | | | | |
| Rehabilitation exercise classes |  | | | |  | | | | |
| Classes for cardiology-related exercises |  | | | |  | | | | |
| Rehabilitation centres |  | | | |  | | | | |
| Physicians’ assistants outside of your practice |  | | | |  | | | | |
| Psychologists |  | | | |  | | | | |
| Medical specialists: Respiratory physicians |  | | | |  | | | | |
| Medical specialists: Internists *(all fields except cardiology)* |  | | | |  | | | | |
| Industrial physicians |  | | | |  | | | | |
| Other, please specify: |  | | | |  | | | | |
|  |  | | | |  | | | | |
|  |  | | | |  | | | | |
| 2.2 Are the general practitioners you cooperate with the same ones as they were 5 years ago? | They are  exactly the same | |  | | | | | There were big  changes | |
|  |  |  | |  | | |  | |  |

| 2.3 Which of the following information on the patient do your medical reports to the general practitioner normally contain?  (Multiple choices possible) | | Diagnoses  ICD-Codes  Anamnesis  Pre-medication  Laboratory results  Instrument-based diagnostics and findings  Status  Summed up evaluation  Therapy suggestions  Other (please specify): __________________________ | | | | | | |
| --- | --- | --- | --- | --- | --- | --- | --- | --- |
| 2.4 How do you handle transmission of results to the general practitioners?  (Multiple choices possible) | | Postal | | | | Via digital medical reports | | |
|  |  | Via phone | | | | Transmission of medical reports via the patient | | |
|  |  | Via fax | | | | Other: _________________ | | |
| 2.5 Which of the following information are mainly included in the general practitioner’s referrals and reports to you? | | | | | | | | |
|  | Never included | | |  | | | | Always included |
| Diagnoses (presumption, by exclusion or confirmed) |  | |  | |  | |  |  |
| Relevant, pre-existing conditions & comorbidities |  | |  | |  | |  |  |
| Anamnesis |  | |  | |  | |  |  |
| Specific question |  | |  | |  | |  |  |
| Somatic status |  | |  | |  | |  |  |
| Allergies/intolerances |  | |  | |  | |  |  |
| Medication |  | |  | |  | |  |  |
| Laboratory results |  | |  | |  | |  |  |
| Diagnostic reports |  | |  | |  | |  |  |

Part 3: Factors in the health care system influencing health care

| 3.1 Care for patients is not solely dependent on the disease or the treating physician. How strong do you think the following aspects influence well-regulated care for patients with chronic cardiovascular diseases in your practice? | | | | | | | |
| --- | --- | --- | --- | --- | --- | --- | --- |
|  | Not at all | |  | | | Very strong | |
| Financial incentives |  |  | |  |  | |  |
| Own practice’s infrastructure |  |  | |  |  | |  |
| Qualification of health care professions |  |  | |  |  | |  |
| Patients’ requests |  |  | |  |  | |  |
| (Selective) contracts (e.g. GP-centred care, medical specialist’s programmes, DMPs) |  |  | |  |  | |  |
| Fear for loss of patients due to them deciding for another physician. |  |  | |  |  | |  |
| Own, subjective competences |  |  | |  |  | |  |
| Other (please specify): |  | | | | | | |
|  |  |  | |  |  | |  |
|  |  |  | |  |  | |  |

Part 4: Statements on cardiology care

Hereafter you find exemplary statements related to cardiology care. Please indicate to what degree you agree with each statement.

|  | Do not agree  at all | |  | | | Agree  completely | | I do not have an opinion on this |
| --- | --- | --- | --- | --- | --- | --- | --- | --- |
| 4.1 Patients with coronary heart disease have to be treated with statins in high dosages. |  |  | |  |  | |  |  |
| 4.2 All patients in a GP’s practice who suffer from dyspnoea and suspected heart failure need to have their BNP-parameters checked. |  |  | |  |  | |  |  |
| 4.3 Every patient with a coronary heart disease and hypertension should reach a systolic target value below 130 mmHg. |  |  | |  |  | |  |  |

Thank you once more for your participation!
