# Supplementary material for: Cooperation networks of ambulatory health care providers: exploration of mechanisms that influence coordination and uptake of recommended cardiovascular care (ExKoCare): a mixed-methods study protocol
Source: BMC Fam Pract. 2020 Aug 16;21:168. doi: 10.1186/s12875-020-01229-3 (PMC7429883; doi:10.1186/s12875-020-01229-3)
Supplement: Supplementary file 2 — Additional file 2. Questionnaire_GPs_provider. [file 12875_2020_1229_MOESM2_ESM.doc]

Questionnaire for General Practitioners (GPs) and non-medic health care professions in the GP’s practice

[translated by the authors from German to English, translation not validated]

# Part 1: General questions

Please check the answer that matches you.

| 1.1 Year of birth | |___|___|___|___| | | | |
| --- | --- | --- | --- | --- |
| 1.2 Sex | Female | Male | | Not specified |
| 1.3 What is your activity status?  Please be aware that this includes any paid/income-related work. | Working full time (35 hours per week or more)  Working part time (less than 35 hour per week) | | | |
| 1.4 Which occupational group do you belong to? | Physicians | | Nurses | |
|  | Physicians’ assistants | | Physiotherapists | |
|  | Psychologists | | | |
|  | Other: ______________________________ | | | |

If you are not a physician, please skip this part and continue with question 2.1.

| 1.5 In which year did you take up residence?  (Please insert year) | |___|___|___|___| |
| --- | --- |
| 1.6 You are a medical specialist for: | General medicine (general practitioner)  Internist working as a general practitioner  Other: _______________________________ |
| 1.7 Do you have additional medical qualifications or primary focuses in cardiovascular areas? | No  Yes, the following:   1. __________________________________ 2. __________________________________ 3. __________________________________ 4. __________________________________ |

Part 2: Questions on information exchange within your practice’s team

Hereafter, please indicate all colleagues and employees within your practice, with which you normally exchange information on patients at least once a week. This encompasses counselling and treating individual patients with (1) Type 2 diabetes, (2) coronary heart disease and (3) chronic heart failure.

The name tags will be removed before the data is analysed, so we only see a pseudonym and not the name. You also may remove the name tags yourself.

| 2.1 Persons within your practice | Type 2 diabetes | | | Coronary heart disease | | | Chronic heart failure | |
| --- | --- | --- | --- | --- | --- | --- | --- | --- |
|  |  | | |  | | |  | |
|  |  | | |  | | |  | |
|  |  | | |  | | |  | |
|  |  | | |  | | |  | |
| 2.2 Are the persons you are working with in this practice the same ones as they were 5 years ago? | They are  exactly the  same | |  | | | | There were big  changes | |
|  |  | | |  |  | |  |

Please indicate the cardiologists you exchange information regarding patients with on a regular base (at least one per month). This encompasses counselling and treatment of patients with (1) coronary heart disease and (2) chronic heart failure.

| 2.3 Persons outside of your practice | Coronary heart disease | Chronic heart failure |
| --- | --- | --- |
|  |  |  |
|  |  |  |
|  |  |  |
|  |  |  |
|  |  |  |
|  |  |  |
|  |  |  |
|  |  |  |
|  |  |  |
|  |  |  |
| Other: |  |  |
| Other: |  |  |
| Other: |  |  |

| 2.4 Are the cardiologists you work together with the same ones as they were 5 years ago? | They are exactly the same |  | | | There were big changes |
| --- | --- | --- | --- | --- | --- |
|  |  |  |  |  |

If you are not a physician, please skip this part and continue with question 2.9.

| 2.5 Which of the following information on the patient do you include in your referral letters to the cardiologist when there are new problems with a patient (no referrals for known diseases)  (Multiple choices possible) | | Diagnoses (presumption, by exclusion or confirmed)  Relevant, pre-existing conditions and comorbidities  Anamnesis  Specific question  Somatic status  Allergies/intolerances  Medication  Laboratory results  Diagnostic reports (e.g. by other physicians)  None of the above | | | | | | |
| --- | --- | --- | --- | --- | --- | --- | --- | --- |
| 2.6 How do you handle transmission of existing results to the cardiologist? | | Digitally | | | | Paper based | | |
|  |  | Partly digital, partly paper based | | | | | | |
| 2.7 Which of the following information are mainly included in the cardiologists’ diagnostic reports to you? | | | | | | | | |
|  | Never included | | |  | | | | Always included |
| Diagnoses |  | |  | |  | |  |  |
| ICD-Codes |  | |  | |  | |  |  |
| Anamnesis |  | |  | |  | |  |  |
| Pre-medication |  | |  | |  | |  |  |
| Laboratory results |  | |  | |  | |  |  |
| Instrument-based diagnostics and findings |  | |  | |  | |  |  |
| Status |  | |  | |  | |  |  |

|  | Never included |  | | | Always included |
| --- | --- | --- | --- | --- | --- |
| Summed up evaluation |  |  |  |  |  |
| Therapy suggestions |  |  |  |  |  |
| Other:  ___________________ |  |  |  |  |  |
| 2.8 After your patient’s appointment with the cardiologist, when do you typically receive the cardiologist’s report? | On the same day  Within 3 days  Within 1 week  Within 2 weeks or more | | | | |

Please indicate the occupational groups **outside** of your practice, with which you exchange information at least once a week on patients with (1) chronic heart failure, (2) coronary heart disease and (3) Type 2 diabetes. This exchange encompasses counselling and treating individual patients (e.g. via different ways of communication like prescriptions, letters, phone calls)

| 2.9 Persons outside of your practice | Type 2 diabetes | Coronary heart disease | Chronic heart failure |
| --- | --- | --- | --- |
| Pharmacists |  |  |  |
| Nutritionists |  |  |  |
| Physiotherapists |  |  |  |
| Nurses in a nursing home |  |  |  |
| Ambulatory nursing services |  |  |  |
| Rehabilitation exercise classes |  |  |  |
| Classes for cardiology-related exercises |  |  |  |
| Rehabilitation centres |  |  |  |
| Physicians’ assistants outside of your practice |  |  |  |

|  | Type 2 diabetes | | | Coronary heart disease | | | Chronic heart failure | | |
| --- | --- | --- | --- | --- | --- | --- | --- | --- | --- |
| Physicians’ assistants outside of your practice |  | | |  | | |  | | |
| Psychologists |  | | |  | | |  | | |
| Medical specialists: Respiratory physicians |  | | |  | | |  | | |
| Medical specialists: Internists *(all fields except cardiology)* |  | | |  | | |  | | |
| Industrial physicians |  | | |  | | |  | | |
| Other, please specify: |  | | | | | | | | |
|  |  | | |  | | |  | | |
|  |  | | |  | | |  | | |
| 2.10 Are the occupational groups you cooperate with the same ones as they were 5 years ago? | They are exactly the same | |  | | | | | There were big changes | |
|  |  | | |  |  | | |  |

**Part 3: Opinions on cardiovascular problems**

|  | Completely disagree | |  | | | Completely agree | |
| --- | --- | --- | --- | --- | --- | --- | --- |
| 3.1 My opinion on cardiovascular problems seems to be unimportant for colleagues. |  |  | |  |  | |  |
| 3.2 When colleagues seek advice on cardiovascular problems, the do not ask me. |  |  | |  |  | |  |
| 3.3 Colleagues often approach me to get advice on cardiovascular problems. |  |  | |  |  | |  |
| 3.4 Colleagues base decisions on cardiovascular problems on my advice. |  |  | |  |  | |  |

|  | Completely disagree |  | | | Completely agree |
| --- | --- | --- | --- | --- | --- |
| 3.5 I often convince colleagues to follow my approach when it comes to cardiovascular problems. |  |  |  |  |  |
| 3.6 I often influence colleagues‘ view on cardiovascular problems. |  |  |  |  |  |
| 3.7 When it comes to cardiovascular problems, I ask colleagues for advice. |  |  |  |  |  |
| 3.8 I do not need exchange with colleagues to make decisions on cardiovascular problems. |  |  |  |  |  |
| 3.9 I rarely ask colleagues on how to proceed on cardiovascular problems. |  |  |  |  |  |
| 3.10 I like to hear colleagues‘ opinions before I make decisions on cardiovascular problems. |  |  |  |  |  |
| 3.11 I feel better with making decisions on cardiovascular problems when I heard colleagues‘ opinions before. |  |  |  |  |  |
| 3.12 When I make a decision on cardiovascular problems, colleagues‘ opinions are not important to me. |  |  |  |  |  |
| 3.13 Is there currently an expert who shapes your opinion on cardiovascular diseases? | Yes, a physician within this practice  Yes, a GP outside of this practice  Yes, a cardiologist outside of this practice  Yes, someone else outside of this practice  No, I cannot name such a person | | | | |

Please indicate how strongly you spontaneously agree with each of the following statements on cardiovascular topics.

|  | Do not agree at all | |  | | | Agree completely | | I do not have an opinion on this |
| --- | --- | --- | --- | --- | --- | --- | --- | --- |
| 3.14 Patients with coronary heart disease have to be treated with statins in high dosages. |  |  | |  |  | |  |  |
| 3.15 All patients in a GP’s practice who suffer from dyspnoea and suspected heart failure need to have their BNP-parameters checked. |  |  | |  |  | |  |  |
| 3.16 Every patient with a coronary heart disease and hypertension should reach a systolic target value below 130 mmHg. |  |  | |  |  | |  |  |

Part 4: Cooperation in your practice

We would like to learn something about cooperation in your practice. Team and Team member refer to all physicians and non-medic health care profession in the practice

|  | Not at all | |  | | | | Very strongly |
| --- | --- | --- | --- | --- | --- | --- | --- |
| 4.1 How strongly do you agree with the goals of your team? |  |  | |  |  |  | |
| 4.2 To what degree do you believe other team members have a clear understanding of your team’s goals? |  |  | |  |  |  | |
| 4.3 To what degree do you believe your team’s goals can actually be achieved? |  |  | |  |  |  | |
| 4.4 In your opinion, how worthwhile are these goals for your organisation? |  |  | |  |  |  | |
| 4.5 We have a ‘We’ feeling. |  |  | |  |  |  | |
| 4.6 Team members keep each other up-to-date about work-related matters. |  |  | |  |  |  | |

|  | Not at all | |  | | | | Very strongly |
| --- | --- | --- | --- | --- | --- | --- | --- |
| 4.7 Team members feel understood and accepted by each other. |  |  | |  |  |  | |
| 4.8 Genuine efforts are made to share information in the whole team. |  |  | |  |  |  | |
| 4.9 Are the members of your team willing to question fundamental aspects of their work? |  |  | |  |  |  | |
| 4.10 Does your team critically reflect on potential weaknesses in order to reach their full potential? |  |  | |  |  |  | |
| 4.11 Do team members build on each other’s ideas to reach the best possible results? |  |  | |  |  |  | |
| 4.12 Team members are always looking for fresh, new ways to look at problems. |  |  | |  |  |  | |
| 4.13 In our team we take the time it needs to develop new ideas. |  |  | |  |  |  | |
| 4.14 Team members collaborate together to support the development and implementation of new ideas. |  |  | |  |  |  | |

**4.15 Is there something else you would like to add regarding your perception of and experience with cooperation in your practice’s team (physicians and other health care professions)?**

|  |
| --- |

Part 5: Factors in the health care system influencing health care

| 5.1 Care for patients is not solely dependent on the disease or the treating physician. How strong do you think the following aspects influence well-regulated care for patients with chronic cardiovascular diseases in your practice? | | | | | | | |
| --- | --- | --- | --- | --- | --- | --- | --- |
|  | Not at all |  | | | | | Very strong |
| Financial incentives |  | |  |  |  |  | |
| Own practice’s infrastructure |  | |  |  |  |  | |
| Qualification of health care professions |  | |  |  |  |  | |
| Patients’ requests |  | |  |  |  |  | |
| (Selective) contracts (e.g. GP-centred care, medical specialist’s programmes, DMPs) |  | |  |  |  |  | |
| Fear for loss of patients due to them deciding for another physician. |  | |  |  |  |  | |
| Own, subjective competences |  | |  |  |  |  | |
| Other (please specify): |  | | | | | | |
|  |  | |  |  |  |  | |
|  |  | |  |  |  |  | |

Thank you once more for your participation!
