# Supplementary material for: Cooperation networks of ambulatory health care providers: exploration of mechanisms that influence coordination and uptake of recommended cardiovascular care (ExKoCare): a mixed-methods study protocol
Source: BMC Fam Pract. 2020 Aug 16;21:168. doi: 10.1186/s12875-020-01229-3 (PMC7429883; doi:10.1186/s12875-020-01229-3)
Supplement: Supplementary file 3 — Additional file 3. Questionnaire_GPs_practices. [file 12875_2020_1229_MOESM3_ESM.docx]

Questionnaire on general practitioners‘ practices

[translated by the authors from German to English, translation not validated]

Part 1: Questions on your practice

| 1.1 What type of practice is your practice? | Individual practice | | Group practice | |
| --- | --- | --- | --- | --- |
|  | Shared practice | | Ambulatory health care centre | |
| 1.2 Is your practice part of a practice network? | Yes, of the Go-Lu Ärztenetz Ludwigshafen e.G.  Yes, of the WoGE – Wormser Gesundheitsnetz  Yes, of the Ärztenetz Mittelbaden eG  Yes, of the Ärztenetz Reutlingen  Yes, of the Gesundes Kinzigtal GmbH  Yes, of the Gesundheitsprojekt Mannheim e.G.  Yes, of the G’sundregion Alb-Donau/Ulm  No | | | |
| 1.3 Are there non-medic employees for intensified case management or case managers in the context of the PracMan programme?  (Multiple choices possible) | Yes, for patients with coronary heart disease  Yes, for patients with chronic heart failure  Yes, for patients with diabetes  Yes, for other patients  No | | | |
| 1.4 Does your practice participate in one or several programmes for GP-centred care?  (Multiple choices possible) | No  Yes, in the following programmes: | | | |
|  | Baden-Württemberg  AOK  LKK  IKK classic  Bosch BKK  BKK VAG  Substitute fund  BKK GWQ  Knappschaft Bahn und See | Rhineland-Palatinate  AOK  LKK  IKK classic  BKK LV Mitte  Techniker  DAK  Other substitute funds  BKK GWQ  spectrum K-BKKen  Axa and Gothaer | | Saarland  IKK classic  Techniker  Substitute funds  BKK GWQ  spectrum K-BKKen |

| 1.5 In which of the following disease-management-programmes (DMP) does your practice participate?  (Multiple choices possible) | Type 2 diabetes | | Heart failure | |
| --- | --- | --- | --- | --- |
|  | Coronary heart disease | | Breast cancer | |
|  | Type 1 diabetes | | Bronchial asthma | |
|  | Chronic obstructive pulmonary disease | | | |
|  | None of them | | | |
| 1.6 How many individual patients do you see per quarter? | < 500  500 – 1000 | | 1001-1500  > 1500 | |
| 1.7 Do you train physician’s assistants in your practice? | Yes | | No | |
| 1.8 Do you train medical specialists in your practice? | Yes | | No | |
| 1.9 Is your practice a training practice for medical students? | Yes | | No | |
| 1.10 How many team meetings (physicians & physicians’ assistants) are there in your practice per quarter? | None  Three | One  Four | | Two  More than four |
| 1.11 How do you managed documentation in your practice? | Digitally | | Paper-based | |
|  | Partly digital, partly paper-based | | | |
| 1.12 How does your practice exchange information with medical specialists’ practices for cardiology?  (Multiple choices possible) | Postal | | Via digital medical reports | |
|  | Via phone | | Transmission of medical reports via the patient | |
|  | Via Fax | | Other: _________________ | |

Part 2: Your team

Please name the colleagues and employees **in your practice** hereinafter. This is necessary to generate he individual questionnaires for your team. All names will be processed separate from the questionnaires, treated confidentially and replaced by a pseudonym for analyses.

| Last name | First name | Function |
| --- | --- | --- |
|  |  | Physician  Physician’s assistant  Other: ______________________ |
|  |  | Physician  Physician’s assistant  Other: ______________________ |
|  |  | Physician  Physician’s assistant  Other: ______________________ |
|  |  | Physician  Physician’s assistant  Other: ______________________ |
|  |  | Physician  Physician’s assistant  Other: ______________________ |
|  |  | Physician  Physician’s assistant  Other: ______________________ |
|  |  | Physician  Physician’s assistant  Other: ______________________ |
|  |  | Physician  Physician’s assistant  Other: ______________________ |

Thank you once more for your participation!
