# Supplementary material for: Cooperation networks of ambulatory health care providers: exploration of mechanisms that influence coordination and uptake of recommended cardiovascular care (ExKoCare): a mixed-methods study protocol
Source: BMC Fam Pract. 2020 Aug 16;21:168. doi: 10.1186/s12875-020-01229-3 (PMC7429883; doi:10.1186/s12875-020-01229-3)
Supplement: Supplementary file 4 — Additional file 4. Questionnaire_patients. [file 12875_2020_1229_MOESM4_ESM.docx]

Questionnaire for patients

[translated by the authors from German to English, translation not validated]

# Part 1: General questions

Please insert or check the answer that matches you

| 1.1 Year of birth | \|___\|___\|___\|___\| |
| --- | --- |
| 1.2 Sex | Female  Male  Not specified |
| 1.3 What is your activity status?  Please be aware that this includes any paid/income-related work. | Not working (retired, student, unemployed)  Working full time (35 hours per week or more)  Working part time (less than 35 hour per week)  Maternity/parental leave  Trainee  Not specified |
| 1.4 Do you have German citizenship? | Yes  No  Not specified |
| 1.5 Do your parents have German citizenship? | Both parents have German citizenship  One parent has German citizenship  None of them has German citizenship  Not specified |
| 1.6 Which type of health insurance are you with? | Statutory Health Insurance  Private Health Insurance  Self-pay patient  Other: __________________________________________ |

Part 2: Questions on your medical history

Please check the answer that matches you.

| 2.1 Because of which heart or circulation disease(s) are you currently treated? (Multiple choice possible) | High blood pressure (hypertension)  Cardiac arrhythmia/atrial fibrillation  Coronary heart disease (incl. angina pectoris and heart attack)  Chronic heart failure  Stroke  Peripheral artery occlusive disease  Aortic aneurysm  Other: _____________________________ | | |
| --- | --- | --- | --- |
| 2.2 Besides a heart or circulation condition, do you suffer from additional, chronic diseases? | No, I do not suffer from additional, chronic diseases  Yes, (multiple choice possible)  Arthropathy (e.g. arthrosis, rheumatism)  Chronic back pain  Type 2 diabetes  Chronic renal disease  Chronic pulmonary disease (e.g. asthma or COPD)  Chronic thyroid disease  Chronic gastrointestinal disease  Allergy/chronic skin disease  Depression or pathological fears  Cancer  Other: _____________________________ | | |
| 2.3 Do you participate in a programme for general practitioner-centred care? | Yes | No | I don’t know |
| 2.4 In which disease-management-programmes (DMP) do you participate? (Multiple choices possible) | DMP type 2 diabetes  DMP heart failure  DMP coronary heart disease  DMP breast cancer  DMP Type 1 diabetes  DMP bronchial asthma  DMP chronic obstructive pulmonary disease (COPD)  I do not participate in a DMP | | |

Part 3: Health care

We are interested in your experiences and impressions regarding health care in the past 12 months. For each statement, please indicate the answer that suits your opinion best.

| The following statements refer to your own general practitioner (GP)  Your own GP is the physician that you typically consult at first in case of a disease.  If you did not see your GP in the past 12 months, please continue with question 3.9. | | | | | | |
| --- | --- | --- | --- | --- | --- | --- |
|  | Completely correct | Correct | Neutral | Incorrect | Completely incorrect | Don’t know/ not sure |
| 3.1 I know my GP very well. |  |  |  |  |  |  |
| 3.2 My GP knows my medical history very well. |  |  |  |  |  |  |
| 3.3 My general practitioner always remembers, what he/she did on my last visit. |  |  |  |  |  |  |
| 3.4 My GP knows my familial circumstances very well. |  |  |  |  |  |  |
| 3.5 My GP knows very well what I do in my everyday life. |  |  |  |  |  |  |
| 3.6 If necessary, my GP gets in touch with me without me having to ask him/her for it. |  |  |  |  |  |  |
| 3.7 My GP knows very well what I deem important when it comes to my treatment. |  |  |  |  |  |  |
| 3.8 My GP keeps in touch sufficiently when I’m treated by other medical care providers (e.g. physiotherapists). |  |  |  |  |  |  |

| The following statements refer to the cooperation between care providers in your GP’s practice (e.g. between the GP and the physician’s assistant or between several GPs)  If this section does not apply to you, please continue with question 3.13. | | | | | | |
| --- | --- | --- | --- | --- | --- | --- |
|  | Completely correct | Correct | Neutral | Incorrect | Completely incorrect | Don’t know/ not sure |
| 3.9 These care providers exchange information very well. |  |  |  |  |  |  |
| 3.10 These care providers cooperate very well. |  |  |  |  |  |  |
| 3.11 Care by the care providers is linked together very well. |  |  |  |  |  |  |
| 3.12 The care providers always know very well, what the other care providers have done. |  |  |  |  |  |  |

| The following statements refer to your own cardiologist.  Your own cardiologist is the one who mainly deals with the treatment of your heart/circulation diseases.  If you did not see a cardiology within the past 12 months, please continue with question 3.21. | | | | | | |
| --- | --- | --- | --- | --- | --- | --- |
|  | Completely correct | Correct | Neutral | Incorrect | Completely incorrect | Don’t know/ not sure |
| 3.13 I know this cardiologist very well. |  |  |  |  |  |  |
| 3.14 My cardiologist knows my medical history very well. |  |  |  |  |  |  |
| 3.15 My cardiologist always remembers, what he/she did on my last visit. |  |  |  |  |  |  |
| 3.16 My cardiologist knows my familial circumstances very well. |  |  |  |  |  |  |
| 3.17 My cardiologist knows very well what I do in my everyday life. |  |  |  |  |  |  |
| 3.18 If necessary, my cardiologist gets in touch with me without me having to ask him/her for it. |  |  |  |  |  |  |
| 3.19 My cardiologist knows very well what I deem important regarding my care. |  |  |  |  |  |  |
| 3.20 This cardiologist stays in touch sufficiently when it comes to my treatment. |  |  |  |  |  |  |

| The following statements refer to the cooperation between your GP and your cardiologist.  If this section does not apply to you, please continue with question 4.1. | | | | | | |
| --- | --- | --- | --- | --- | --- | --- |
|  | Completely correct | Correct | Neutral | Incorrect | Completely incorrect | Don’t know/ not sure |
| 3.21 These care providers exchange information very well. |  |  |  |  |  |  |
| 3.22 These care providers cooperate very well. |  |  |  |  |  |  |
| 3.23 Care by the care providers is linked together very well. |  |  |  |  |  |  |
| 3.24 These care providers always know very well, what the other care providers have done. |  |  |  |  |  |  |

Part 4: Contact with physicians and other health care professions

We are interested in the number of contacts you had with physicians and other care providers during the past **3 months**.

| 4.1 With how many GPs are you regularly in touch?  This encompasses every kind of contact connected to the provision of medical services (e.g. examinations, treatments, ordering and fetching prescriptions, requesting results).  Purely administrative/organisational contacts (scheduling appointments, questions on opening hours, etc.) do not count. | Only with (my) one GP  With several GPs. Number: \|__\|__\| | | |
| --- | --- | --- | --- |
| 4.2 During the past 3 months, how often were you personally in touch (in person or via phone) with your GP or your GP’s practice in total?  This encompasses every kind of contact connected to the provision of medical services (e.g. examinations, treatments, ordering and fetching prescriptions, requesting results).  Purely administrative/organisational contacts (scheduling appointments, questions on opening hours, etc.) do not count. | \|__\|__\|__\| times | | |
| 4.3 During the past 3 months, how often did you visit your GP’s consultation-hour? | \|__\|__\|__\| times | | |
| 4.4 Is your heart/circulation disease treated by one or more cardiologists? | By one cardiologist  By several cardiologists. Number: \|__\|__\| | | |
| 4.5 During the past 3 months, how often were you personally in touch (in person or via phone) with your cardiologist or your cardiologist’s practice in total?  This encompasses every kind of contact connected to the provision of medical services (e.g. examinations, treatments, ordering and fetching prescriptions, requesting results).  Purely administrative/organisational contacts (scheduling appointments, questions on opening hours, etc.) do not count. | \|__\|__\|__\| times | | |
| 4.6 During the past 3 months, how often did you visit your cardiologist’s consultation-hour? | \|__\|__\|__\| times | | |
| 4.7 In the past 3 months, with which of the following physicians and/or other health care providers were you in touch just because of your heart/circulation disease? And how often were you in touch because of it?  This encompasses every kind of contact connected to the provision of medical services (e.g. examinations, treatments, ordering and fetching prescriptions, requesting results).  Purely administrative/organisational contacts (scheduling appointments, questions on opening hours, etc.) do not count. | | | |
|  | | In the past 3 months, was there any contact because of your heart/circulation disease? | Number of contacts in the past 3 months (Please insert) |
| GP | | Yes  No | \|__\|__\|__\| |
| Physician’s assistant in the GP’s practice | | Yes  No | \|__\|__\|__\| |
| Cardiologist | | Yes  No | \|__\|__\|__\| |
| Physician’s assistant in the cardiologist’s practice | | Yes  No | \|__\|__\|__\| |
| Other medical specialists’ practices | | Yes  No | \|__\|__\|__\| |
| Classes for cardiology-related exercises | | Yes  No | \|__\|__\|__\| |

|  | In the past 3 months, was there any contact because of your heart/circulation disease? | Number of contacts in the past 3 months (Please insert) |
| --- | --- | --- |
| Pharmacy | Yes  No | \|__\|__\|__\| |
| Chiropody practices | Yes  No | \|__\|__\|__\| |
| Ambulatory nursing services | Yes  No | \|__\|__\|__\| |
| Other: | Yes  No | \|__\|__\|__\| |
| Other: | Yes  No | \|__\|__\|__\| |

Part 5: Constitutional measures and therapies

We are interested in the preventive and constitutional measures you took part in during the past 12 months because of your heart disease.

| 5.1 How often do you perform physical activities that leave you at least a little out of breath or make you sweat (e.g. sports, hiking, dancing, gardening, etc.)? | Daily  3 to 6 times a week  1 to 2 times a week  Less regularly, about once a month  Never |
| --- | --- |
| 5.2 Did your GP advice you on exercises during the past 12 months? | Yes  No |
| 5.3 Did your GP advice you on your smoking habits during the past 12 months? | Yes  No  I don’t smoke |
| 5.4 Did your GP advice you on your drinking habits during the past 12 months? | Yes  No  I don’t drink alcohol |
| 5.5 Did your GP check your body weight and advice you accordingly during the past 12 months? | Yes  No |

| 5.6 Did you talk to you GP about your eating habits during the past 12 months? | Yes  No |
| --- | --- |
| 5.7 Did you receive information material on your heart disease during the past 12 months? | Yes  No |
| 5.8 Did you agree therapeutic goals together with your GP during the past 12 months? | Yes  No |
| 5.9 Did you take a statin (to lower cholesterol) during the past 12 months? | Yes  No |
| 5.10 Do you have a medication plan and did you talk about it with your GP at least once during the past 12 months? | Yes, I have a medication plan and talked about it with my GP.  Yes, I have a medication plan and did not talk about it with my GP  No |

We are very thankful for your participation in this research project!
