# Supplementary material for: Cooperation networks of ambulatory health care providers: exploration of mechanisms that influence coordination and uptake of recommended cardiovascular care (ExKoCare): a mixed-methods study protocol
Source: BMC Fam Pract. 2020 Aug 16;21:168. doi: 10.1186/s12875-020-01229-3 (PMC7429883; doi:10.1186/s12875-020-01229-3)
Supplement: Supplementary file 5 — Additional file 5. NCQ_german. [file 12875_2020_1229_MOESM5_ESM.docx]

Nijmegen Continuity Questionnaire

[translated by the authors from English to German, translation not validated]

Wir sind an Ihren Erfahrungen und Eindrücken bezüglich Ihrer Gesundheitsversorgung in den letzten 12 Monaten interessiert. Bitte kreuzen Sie bei jeder der folgenden Aussagen die Antwort an, die am besten Ihrer Meinung entspricht.

| Die folgenden Aussagen beziehen sich auf Ihren eigenen Hausarzt  Der eigene Hausarzt ist derjenige Arzt, den Sie bei einer Erkrankung in der Regel als erstes aufsuchen.  Wenn Sie Ihren Hausarzt im vergangenen Jahr nicht gesehen haben, fahren Sie bitte mit dem nächsten Abschnitt fort. | | | | | | |
| --- | --- | --- | --- | --- | --- | --- |
|  | Stimmt völlig | Stimmt | Neutral | Stimmt nicht | Stimmt gar nicht | Weiß nicht/ Unklar |
| 3.1 Ich kenne meinen Hausarzt sehr gut. |  |  |  |  |  |  |
| 3.2 Mein Hausarzt kennt meine Krankengeschichte sehr gut. |  |  |  |  |  |  |
| 3.3 Mein Hausarzt weiß immer sehr gut, was er/sie zuvor getan hat. |  |  |  |  |  |  |
| 3.4 Mein Hausarzt kennt meine familiären Umstände sehr gut. |  |  |  |  |  |  |
| 3.5 Mein Hausarzt kennt meine täglichen Aktivitäten sehr gut |  |  |  |  |  |  |
| 3.6 Mein Hausarzt kontaktiert mich, wenn nötig, ich muss ihn nicht darum bitten. |  |  |  |  |  |  |
| 3.7 Mein Hausarzt weiß sehr gut, was ich bei meiner Versorgung als wichtig erachte. |  |  |  |  |  |  |
| 3.8 Mein Hausarzt hält ausreichend Kontakt mit mir, wenn ich von anderen medizinischen Leistungserbringern (z.B. Physiotherapeut) betreut werde. |  |  |  |  |  |  |

| Die folgenden Aussagen beziehen sich auf die Kooperation zwischen Leistungserbringern in der Hausarztpraxis (z.B. zwischen dem Hausarzt und der Arzthelferin/Sprechstundenhilfe oder zwischenverschiedenen Hausärzten)  Wenn dieser Abschnitt nicht auf Sie zutrifft fahren Sie bitte mit dem nächsten Abschnitt fort. | | | | | | |
| --- | --- | --- | --- | --- | --- | --- |
|  | Stimmt völlig | Stimmt | Neutral | Stimmt nicht | Stimmt gar nicht | Weiß nicht/Unklar |
| 3.9 Diese Leistungserbringer geben Informationen sehr gut aneinander weiter. |  |  |  |  |  |  |
| 3.10 Diese Leistungserbringer arbeiten sehr gut zusammen. |  |  |  |  |  |  |
| 3.11 Die Versorgung durch diese Leistungserbringer ist gut verbunden. |  |  |  |  |  |  |
| 3.12 Diese Leistungserbringer wissen immer sehr gut, was die anderen jeweils tun. |  |  |  |  |  |  |

| Die folgenden Aussagen beziehen sich auf Ihren eigenen Kardiologen  Der eigene Kardiologe ist der Herzspezialist, der sich überwiegend um die Behandlung ihrer Herz-/Kreislauferkrankung kümmert.  Wenn Sie im letzten Jahr keinen Kardiologen gesehen haben, fahren Sie bitte mit dem nächsten Abschnitt fort. | | | | | | |
| --- | --- | --- | --- | --- | --- | --- |
|  | Stimmt völlig | Stimmt | Neutral | Stimmt nicht | Stimmt gar nicht | Weiß nicht/ Unklar |
| 3.13 Ich kenne diesen Kardiologen sehr gut. |  |  |  |  |  |  |
| 3.14 Dieser Kardiologe kennt meine Krankengeschichte sehr gut. |  |  |  |  |  |  |
| 3.15 Dieser Kardiologe weiß immer sehr gut, was er/sie zuvor getan hat. |  |  |  |  |  |  |
| 3.16 Dieser Kardiologe kennt meine familiären Umstände sehr gut. |  |  |  |  |  |  |
| 3.17 Dieser Kardiologe kennt meine täglichen Aktivitäten sehr gut. |  |  |  |  |  |  |
| 3.18 Dieser Kardiologe kontaktiert mich, wenn nötig, ich muss nicht darum bitten. |  |  |  |  |  |  |
| 3.19 Dieser Kardiologe weiß sehr gut, was ich bei meiner Versorgung als richtig erachte. |  |  |  |  |  |  |
| 3.20 Dieser Kardiologe hält ausreichend Kontakt mit mir, wenn ich von anderen medizinischen Leistungserbringern betreut werde. |  |  |  |  |  |  |

| Die folgenden Aussagen beziehen sich auf die Kooperation zwischen Ihrem Hausarzt und Ihrem Kardiologen.  Wenn dieser Abschnitt nicht auf Sie zutrifft, fahren Sie bitte mit dem nächsten Abschnitt fort. | | | | | | |
| --- | --- | --- | --- | --- | --- | --- |
|  | Stimmt völlig | Stimmt | Neutral | Stimmt nicht | Stimmt gar nicht | Weiß nicht/ Unklar |
| **3.21 Diese Leistungserbringer geben Informationen sehr gut aneinander weiter.** |  |  |  |  |  |  |
| **3.22 Diese Leistungserbringer arbeiten sehr gut zusammen.** |  |  |  |  |  |  |
| **3.23 Die Versorgung durch diese Leistungserbringer ist sehr gut verbunden.** |  |  |  |  |  |  |
| **3.24 Diese Leistungserbringer wissen immer sehr gut, was die anderen Leistungserbringer getan haben.** |  |  |  |  |  |  |
